# Improved hyponatremia after pericardial drainage in patients suffering from cardiac tamponade

**DOI:** 10.1186/s12872-016-0316-1

**Published:** 2016-06-11

**Authors:** Bor-Hsin Jong, Cheng-Chun Wei, Kou-Gi Shyu

**Affiliations:** Division of Cardiology, Department of Internal Medicine, Shin Kong Wu Ho-Su Memorial Hospital, 95, Wen-Chang Road, 11101 Taipei, Taiwan; Graduate Institute of Clinical Medicine, Taipei Medical University, Taipei, Taiwan

## Abstract

**Background:**

Some case reports showed unexplained hyponatremia in patients with cardiac tamponade. Reversible hyponatremia was observed in these patients who received pericardial drainage. The occurrence rate of hyponatremia in patients of cardiac tamponade is not clearly known. The objective of this study was to identify the relationship between hyponatremia, cardiac tamponade and their underlying diseases.

**Methods:**

We reviewed the clinical data of patients with cardiac tamponade and receiving pericardial drainage between January 2000 and January 2012 in our hospital. Cardiac tamponade was diagnosed by clinical presentation: hypotension, pulsus paradoxus, and increased jugular vein pressure. We used paired *T* test to compare the sodium change before and after pericardial drainage. Pearson’s chi-square test was used to analyze the relationship of hyponatremia with malignancy and cardiac chamber compression proved by echocardiography.

**Results:**

For the 48 patients, the mean pre-drainage sodium level was 129.1 ± 7.1 mEq/L and the mean post-drainage sodium level was 130.4 ± 5.6 mEq/L (*p* = 0.06). Among the 48 patients, 31 (65 %) had hyponatremia. For the 31 hyponatremia patients, the mean pre-drainage sodium level was 124.8 ± 4.9 mEq/L and the mean post drainage sodium level was 127.5 ± 4.5 mEq/L (*p* = 0.003). Hyponatremia was significantly associated with malignancy (*p* = 0.038). There was no significant change of pre-drainage and post-drainage sodium level in patients without malignancy. The post-drainage sodium level in the malignant patients significantly increased from 125.5 ± 8.0 to 129.1 ± 5.5 mEq/L (*p* = 0.017). The presence of hyponatremia was strongly associated with the cardiac tamponade sign (*p* < 0.001). After pericardial drainage, the sodium level significantly increased in patients with chamber compression than in patients without compression.

**Conclusion:**

Hyponatremia is associated with cardiac tamponade especially for malignant pericardial effusion and for patients with cardiac chambers compression signs. Hyponatremia can be improved after pericardial effusion drainage.

## Background

Cardiac tamponade is a clinical emergency. The rapid growth of large amount of fluid limits the space of pericardium and further impairs the contractility of heart. Several case reports have shown strong correlation between cardiac tamponade and hyponatremia [1–4]. Hyponatremia is frequently encountered in patients with cardiac tamponade, especially for cancer patients [4, 5]. No common causes of hyponatremia could be found except cardiac tamponade. Interestingly, the hyponatremia would recover spontaneously and rapidly after pericardial drainage without additional salt diet, diuretics use or other management [4]. These observational results made the authors to conclude that cardiac tamponade and hyponatremia were directly related. The authors commented the decreased cardiac output might stimulate antidiuretic hormone release [1–5]. After relief of the pressure in cardiac chambers, the neurohormone would decrease and be accompanied with diuresis which would increase the sodium concentration [1–5].

Most of the hyponatremia in cardiac tamponade was reported by case report [1–4]. Recent study reported by Chang et al. did not have echocardiography data [5]. No systemic data on hyponatremia and cardiac tamponade was shown. The occurrence rate of hyponatremia in patients with cardiac tamponade is not clearly known. Since both hyponatremia and cardiac tamponade are critical conditions, the relationship between hyponatremia and cardiac tamponade should be clearly defined.

In this retrospective study, we collected patients’ clinical data including clinical diagnosis, underlying diseases, electrolytes, vital signs and echocardiography findings and systemically analyzed the relationship of pericardial drainage and the change of sodium concentration in patients with cardiac tamponade.

## Methods

### Patients

We used our electronic database to retrieve cases by international classification of diseases (ICD-9) code 423.0 (hemopericardium), 423.3 (cardiac tamponade), and 423.9 (unspecified disease pericardium) between January 2000 and January 2012 in our hospital. We reviewed the history, vital signs, physical findings, chest radiography, electrolytes data, cardiac echo findings, medications and management of individual case. Cardiac tamponade was diagnosed with clinical presentation. Clinical criteria for cardiac tamponade were as follows: hypotension (systolic blood pressure less than 90 mmHg), pulsus paradoxus (a decline of more than 10 mmHg in inspiratory systolic blood pressure), and increased jugular venous pressure [6].

Hyponatremia was defined as sodium concentration below 135 mEq. Patients undergoing pericardial effusion drainage and having serum sodium concentrations checked before and after drainage within 48 h were enrolled for further analysis. Those who had incomplete electrolytes data or who received additional diet salt for hyponatremia correction or who received diuretics were excluded. The patients who have identified causes of hyponatremia were also excluded. This study was carried out in a single tertiary medical center.

### Echocardiography

Most cases received echocardiography before emergent pericardial drainage. The echocardiography specialists would record the amount of pericardial effusion and the right atrium or right ventricle compression during diastolic phase. The size of inferior vena cava and variation during respiration were also recorded as an adjunct for tamponade diagnosis. The large amount of pericardial effusion was defined as the sum of anterior and posterior free echo space exceeds 20 mm at end diastole [7]. Cardiac chamber compression of right ventricle is an absolute indication for decompression in the study.

Emergent pericardial drainage would also be suggested electively for the patients with rapid growth or large amount of pericardial effusion with unstable clinical conditions. Both cases were enrolled in our series. Pericardial drainage was arranged either by percutaneous pericardiocentesis or surgical drainage based on clinicians’ judgment.

### Statistical analysis

Paired *T* test was used to analyze the sodium change before and after pericardial effusion drainage. The Pearson’s chi-square test was used to analyze the frequency of hyponatremia in patients with cardiac chamber compression comparing with non-cardiac chamber compression and with malignancy comparing with non-malignancy pericardial effusion. A *P* value of less than 0.05 was considered to indicate significance for all other outcomes. All analyses were conducted with the use of SPSS software, version 17.0 (SPSS Inc., Chicago, IL, USA).

## Results

Eighty-six patients were identified from January 2000 to January 2012 in our hospital. Twelve patients were excluded because of lack of paired sodium concentration before and after pericardial effusion drainage; 12 were excluded because of identified causes of hyponatremia; 9 patients were excluded because of additional diet salt or diuretics use; 5 patients were excluded because of lack of complete echocardiography data. 48 patients were enrolled and their characteristics are summarized in Table 1. Fifty-eight percent of them had hypertension, 27 % of them had coronary artery diseases, and 19 % had diabetes mellitus. Half of the patients are male and the mean age is 71 years.

**Table 1:** Patient characteristics

	Number	Percent
Sex		
Men	24	50 %
Women	24	50 %
Age(yrs)	71 ± 13	
Underlying diseases		
Diabetes mellitus	9	19 %
Hypertension	28	58 %
Coronary artery disease	13	27 %
Dyslipidemia	9	19 %
Diagnosis		
Malignancy related	11	23 %
Colorectal cancer	1	2 %
Lung cancer	4	8 %
Thymic carcinoma	1	2 %
Breast cancer	2	4 %
Metastatic cancer with unknown origin	1	2 %
Esophageal cancer	1	2 %
Lymphoma	1	2 %
Benign cause		
Tuberculosis	10	22 %
Uremic pericarditis	6	12 %
Autoimmune	4	8 %
Iatrogenic	9	19 %
Hypothyroidism	3	6 %
Trauma	1	2 %
Hydropericardium	4	8 %

Among all 48 enrolled patients, 11 of them were diagnosed to have malignancy; the acutal cytology of the pericaridial effusion of all 11 patients is listed in Table 2. All of the known etiology of cytology in the pericardial fluid is identical to the proposed underlying malignancy; the only one with metastatic cancer with unknown origin is squamous cell carcinoma. Ten of the 37 patients with no malignancy were diagnosed to have tuberculosis; the pericardial effusions of all 10 patients were tested, and the polymerase train reaction (PCR) of all 10 specimens revealed *Mycobacterium tuberculosis*. Four of the 37 patients with no malignancy were diagnosed to have hydropericardium, which the cytology results of malignancy and PCR of *Mycobacterium tuberculosis* were negative.

**Table 2:** The results of cytology the patients with malignant pericardial drainage

Diagnosis	
Patients with Malignant pericardial effusion	11
Colorectal cancer	1
Patient 1: adenocarcinoma	
Lung cancer	4
Patient 2: adenocarcinoma	
Patient 3: adenocarcinoma	
Patient 4: adenocarcinoma	
Patient 5: adenocarcinoma	
Thymic carcinoma	1
Patient 6: thymic carcinoma	
Breast cancer	2
Patient 7: breast carcinoma	
Patient 8: breast carcinoma	
Metastatic cancer with unknown origin	1
Patient 9: squamous cell carcinoma	
Esophageal cancer	1
Patient 10: esophageal squamous cell carcinoma	
Lymphoma	1
Patient 11: lymphoma	

For our 48 patients, the overall mean pre-drainage sodium level was 129.1 ± 7.1 mEq/L and the mean post-drainage sodium level was 130.4 ± 5.6 mEq/L. The difference is 1.2 ± 4.3 mEq/L (*p* = 0.06). Among the 48 patients, 31(65 %) had hyponatremia. Their mean pre-drainage sodium level was 124.8 ± 4.9 mEq/L and the mean post-drainage sodium level was 127.5 ± 4.5 mEq/L. The post-drainage serum sodium level was significantly higher than the pre-drainage level and the mean increase is 2.6 mEq/L (*p* = 0.003).

**Fig. 1:**
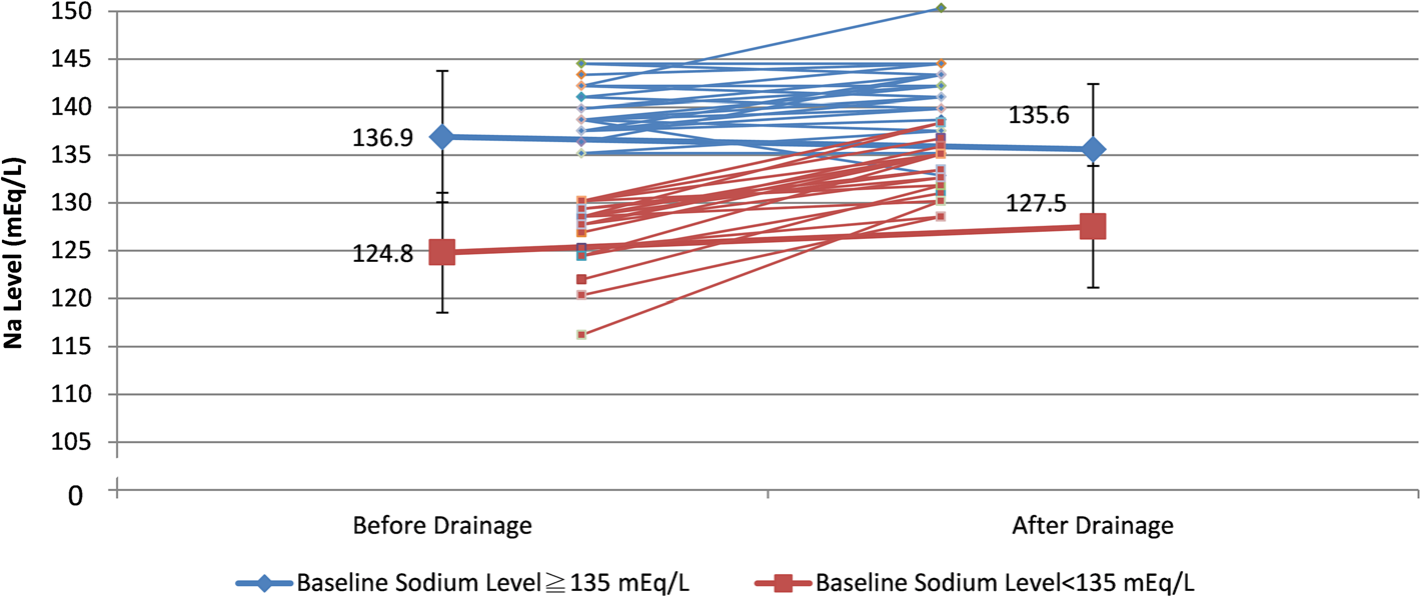
The serum sodium level change before and after drainage and the comparison of change between the group with serum sodium level < 135 mEq/L and the group with serum sodium level ≥135 mEq/L

**Fig. 2:**
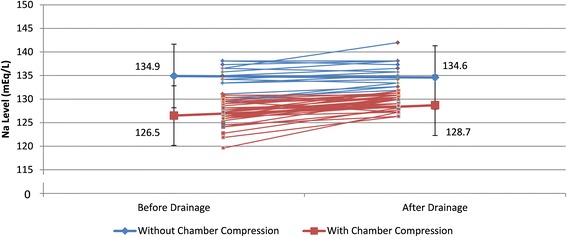
The serum sodium level change before and after drainage and the comparison of change between the group with chamber compression and the group without chamber compressionᅟ

For our 48 patients, 11 suffered from malignant pericardial effusion; 10 of them (91 %) had hyponatremia. Other 37 patients were associated with non-malignancy causes and 21 (57 %) had hyponatremia (Table 3). The sodium level in malignant etiology of tamponade was lower than that of benign etiology of tamponade. Pericardial drainage did not significantly affect the sodium level in patients with higher baseline (Na≧135 mEq/L) but significantly increased sodium level in patients with Na < 135 mEq/L (Fig. 1). The pericardial drainage did not significantly affect sodium level in patients without chamber compression but significantly increased sodium level in patients with chamber compression (Fig. 2).

**Table 3:** The comparison of sodium level between pre and post pericardial drainage

Subgroup	Pericardial drainage			
	Before	After	Difference	*P*
Total (*N* = 48)	129.1 ± 7.1	130.4 ± 5.6	1.2 ± 4.3	0.06
Baseline sodium level				
≧135 mEg/L (*n* = 17)	136.9 ± 2.2	135.6 ± 2.5	1.4 ± 2.6	0.051
< 135 mEg/L (*n* = 31)	124.8 ± 4.9	127.5 ± 4.5	2.6 ± 4.5	0.003
Etiology of tamponade				
Benign (*n* = 37)	130.2 ± 6.6	131 ± 5.6	0.7 ± 4.6	0.321
Malignant (*n* = 11)	125.5 ± 8.0	129.1 ± 5.5	3.6 ± 4.2	0.017
Chamber compression				
None (*n* = 15)	134.9 ± 4.5	134.6 ± 4.0	0.3 ± 2.2	0.571
Yes (*n* = 33)	126.5 ± 6.6	128.7 ± 5.2	2.2 ± 5.2	0.02

Data were presented as mean ± standard deviation

P = paired-student *t*-test

**Fig. 3:**
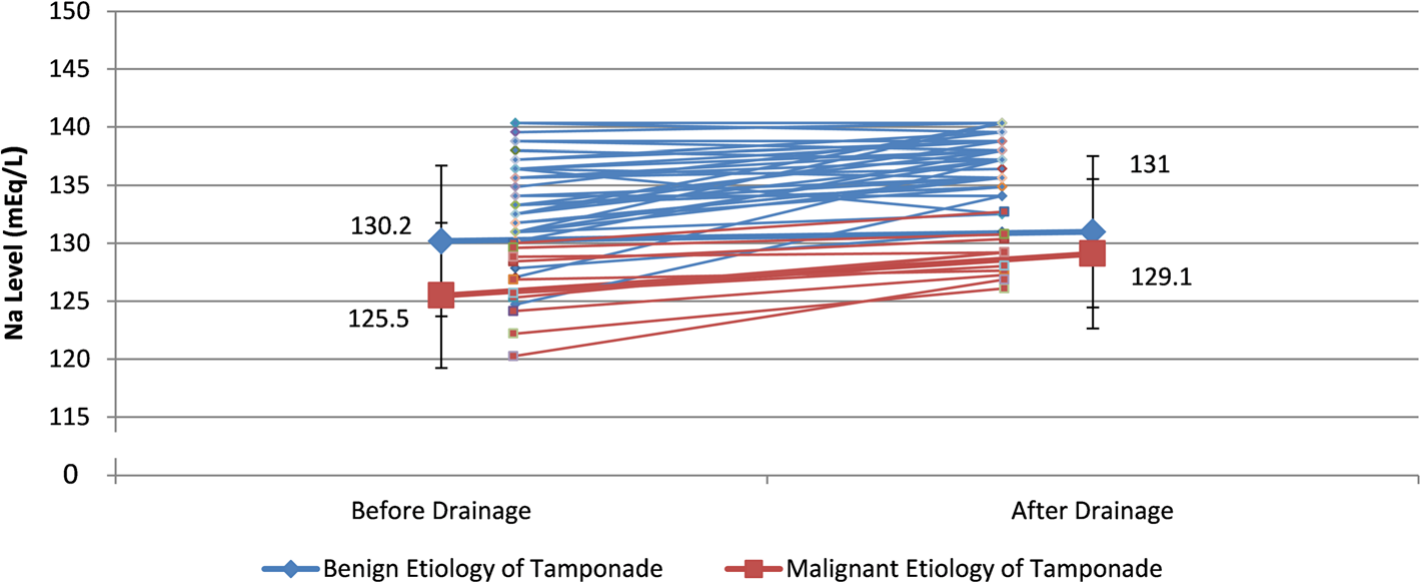
The serum sodium level change before and after drainage and the comparison of change between the group with malignant etiology of tamponade and the group with benign etiology of tamponade

Hyponatremia was significantly associated with malignancy (*p* = 0.038). (Table 3) For our patients, 33 (69 %) patients have cardiac chambers compression proven by echocardiography; 15 (31 %) received pericardial fluid drainage by clinical symptoms and large amount of pericardial effusion. Among the cardiac chambers compression group, 27 of 33(82 %) had hyponatremia. In another clinical diagnosis group, 4 of 15 (27 %) had hyponatremia. The presence of hyponatremia was strongly associated with the cardiac chambers compression signs (*p* < 0.001). (Table 4) After pericardial drainage, the sodium level significantly increased in patients with chamber compression than in patients without chamber compression (Fig. 3).

**Table 4:** The comparison of hyponatremia before pericardial drainage between groups

Subgroup	Hyponatremia at baseline		
	None	Yes	*P*
Etiology of tamponade			0.038
Benign	16 (43 %)	21 (57 %)	
Malignant	1 (36 %)	10 (64 %)	
Chamber compression			<0.001
None	11 (73 %)	4 (27 %)	
Yes	6 (18 %)	27 (82 %)	

Data were number and percentage

The ESC Cardiac Tamponade Score was also calculated of all 48 patients, which average score was 4.28; the average score of the higher baseline of sodium level (Na≧135 mEq/L) was 3.72, whereas the average score of the lower baseline of sodium level (Na < 135 mEq/L) was 4.91 [8] (Table 5).

**Table 5:** The average and SD of the ESC Cardiac Tamponade Score

ESC score	Average	SD
Total (*N* = 48)	4.28	2.76
Baseline sodium level		
≧135 mEg/L (*n* = 17)	3.72	2.46
< 135 mEg/L (*n* = 31)	4.91	3.01

Data were numbers. *SD* standard deviation

## Discussion

Hyponatremia is a common electrolyte disturbance associated with considerable morbidity and mortality [9]. Hyponatremia causes nonspecific symptoms and may be overlooked easily. Nonetheless, it might reflect the severity of underlying diseases [9, 10].

In our study, 65 % of patients were presented with hyponatremia and cardiac tamponade. We considered cardiac tamponade to be one but not the only factors to determine the sodium level in these patients. Undeniable, there still existed some confounding factors in our study. Congestive heart failure, hypothyroidism, acute kidney injury and malignancies are all possible causes for hyponatremia [9]. We could not exclude all these because of small case numbers; besides, they were very common clinical problems in the cardiac tamponade patients. We inferred that tamponade is a possible etiology of hyponatremia because the pericardial drainage recovered the hyponatremia soon and effectively without additional management. Furthermore, we did not check their antidiuretic hormone (ADH) level, or record their daily urine output, osmolality or monitor their intra-cardiac pressure because of high cost and ineffectiveness.

About one third of our patients with hyponatremia had underlying malignancy. In addition, comparing the diagnosis of pericardial effusion, malignancy-related tamponade showed higher rates of hyponatremia than those without malignancy. The severity of hyponatremia and the increase of sodium level after pericardial drainage in the malignancy group were also higher than the non-malignancy group. Malignancy seemed to be a predictor for the development of hyponatremia. The mechanism was unknown but possibly related with tumor secreting factors such as ADH and atrial natriuretic peptide [11–13]. To clarify this, we need a further large clinical trial.

Cardiac chambers compression, especially for ventricles, is a strong indication for pericardial drainage [7]. There was strong positive correlation between hyponatremia and cardiac chambers compression. In contrast to some elective situations that patients underwent pericardial drainage because of acute symptoms and worsening conditions, patients with cardiac compression had higher incidence and severity of hyponatremia. After pericardial drainage, they also had prominent sodium level increase. Therefore, we considered cardiac chambers compression is also a predictor of the development of hyponatremia.

Hyponatremia developing in the setting of cardiac tamponade was rarely reported [1–5]. Mouallem et al. reported a patient with lung cancer complicated with malignant pericardial effusion [4]. Rehan et al. reported an idiopathic pericarditis with large amount of pericardial effusion leading to symptomatic pericardial tamponade [2]. Peter et al. presented a 29-year-old paraplegic man who was being treated with warfarin [3]. Hyponatremia rapidly resolved after pericardiocentesis. These patients’ laboratory data before pericardiocentesis showed high urine osmolality and high urine sodium waste. Removal of pericardial fluid by means of pericardiocentesis resulted in rapid improvement in cardiac output and hemodynamic status. In response to these changes, urine output increased promptly with excretion of large volume of dilute urine and rapid correction of hyponatremia. The authors commented 2 possible hypotheses to explain the relationship of hyponatremia and cardiac tamponade. First, the increased pressure in the compressed cardiac chambers may reactivate the ADH release [5, 6, 13]. The ADH would keep free water retention and cause hyponatremia. Second, the decreased cardiac output may contribute to impairment of urine diluting ability and make the free water clearance much less.

In our study, we did not collect the urine and serum osmolality, urine sodium and the urine amount after pericardiocentesis. It is warranted that further prospective study to investigate the relationship of urine and serum osmolality in patients with cardiac tamponade.

Furthermore, we did not collect the N-terminal pro-B-type natriuretic peptide (NT-proBNP) level. Kim et al. reported that NT-proBNP level may be a useful marker of disease severity in patients suffering from pericardial effusion [14]. In our study there was only few of our patients that NT-proBNP level was checked and the data was insufficient to be analyzed.

We calculated the ESC Cardiac Tamponade score of all 48 patients. There was no differences of the average score of overall and between the higher and lower sodium level. According to the algorithm, any patient with score ≧6 or with compromised hemodynamic status needed to receive emergent pericardial drainage. There was no evidence that hyponatremia was related with high ESC Cardiac Tamponade score [8].

### Study limitation

Hyponatremia can have multifactorial causes. In practice, we cannot workup all possible causes for those patients who need emergent pericardiocentesis, therefore lack of the complete data of pericardial drainage of some of our patients may interfere our analysis. The conclusion we made in this article was inferred from the indirect evidence. A larger and well-designed trial should be conducted.

The chamber compression was defined as the right ventricle was compressed that had detected via echocardiography. Thirty-three of 48 patients met the definition and the other 15 patients did not have compression sign of right ventricle but still received pericardial fluid drainage due to unstable hemodynamics clinically. The relation between compression of right ventricle and hyponatremia was seen in our study but still need further larger and well-designed trial to be conducted.

Furthermore, due to retrospective collection of data, there was no any documentation of intrapericardial pressures before and after pericardial drainage, nor the measurement of left ventricular end-diastolic pressure or pericardial cavity pressure before and after pericardial drainage. There was no data of intrapericardial C-reactive protein or other inflammation markers due to the same reason above.

## Conclusions

Cardiac tamponade should be considered in patients with hyponatremia with unexplained causes. Malignancy and cardiac chambers compression were two predictors for the development hyponatremia in these patients. Hyponatremia will recover spontaneously and rapidly after pericardial drainage without additional management.

## Abbreviations

ADH, antidiuretic hormone; ESC, European Society of Cardiology; NT-proBNP, N-terminal pro-B-type natriuretic peptide; PCR, polymerase train reaction
